# Digital IIR Filters Design Using Differential Evolution Algorithm with a Controllable Probabilistic Population Size

**DOI:** 10.1371/journal.pone.0040549

**Published:** 2012-07-11

**Authors:** Wu Zhu, Jian-an Fang, Yang Tang, Wenbing Zhang, Wei Du

**Affiliations:** 1 College of Information Science and Technology, Donghua University, Shanghai, China; 2 Research Institute for Intelligent Control and System, Harbin Institute of Technology, Harbin, China; 3 Institute of Physics, Humboldt University, Berlin, Germany; 4 Potsdam Institute for Climate Impact Research, Potsdam, Germany; 5 Institute of Textiles and Clothing, The Hong Kong Polytechnic University, Hong Kong, China; Université de Nantes, France

## Abstract

Design of a digital infinite-impulse-response (IIR) filter is the process of synthesizing and implementing a recursive filter network so that a set of prescribed excitations results a set of desired responses. However, the error surface of IIR filters is usually non-linear and multi-modal. In order to find the global minimum indeed, an improved differential evolution (DE) is proposed for digital IIR filter design in this paper. The suggested algorithm is a kind of DE variants with a controllable probabilistic (CPDE) population size. It considers the convergence speed and the computational cost simultaneously by nonperiodic partial increasing or declining individuals according to fitness diversities. In addition, we discuss as well some important aspects for IIR filter design, such as the cost function value, the influence of (noise) perturbations, the convergence rate and successful percentage, the parameter measurement, etc. As to the simulation result, it shows that the presented algorithm is viable and comparable. Compared with six existing State-of-the-Art algorithms-based digital IIR filter design methods obtained by numerical experiments, CPDE is relatively more promising and competitive.

## Introduction

Filtering problem [1], [2] is a widely studied research topic in various fields of control and signal processing. The main objective of filtering is synthesizing and implementing a filter network [3] to modify, reshape, or manipulate the frequency spectrum of a signal according to some desired specifications. As one of the most successful filter networks, the well-known Digital infinite-impulse-response (IIR) filter has been extensively used in many practical systems [4]–[7], such as engineering system, network system, nuclear reactor, biological system, chemical system and electrical networks system. However, it has been recognized now that the IIR filter will generally not guarantee satisfactory performance if its feedback coefficients are chosen not appropriately during the adaptation process [8]. Apart from this disadvantage, the possibility of having a multi-modal and nonlinear error surface is another important design challenge for recursive filters [9], [10]. To improve the robustness, in recent years, many heuristic optimization design methods have been developed, such as simulated annealing (SA) [11], ant colony optimization (ACO) [12], particle swarm optimization (PSO) [13], seeker optimization algorithm (SOA) [14], artificial bee colony (ABC) [15], [16] and differential evolution (DE) [17], etc.

A SA is usually sensitive to its starting point of the search and requires too many function evaluations to converge to the global minima. The ACO imitates the social behavior of real ant colonies and it has been originally developed for combinatorial optimization problems. But, it may occasionally be trapped into local stagnation or premature convergence resulting in a low optimizing precision or even failure [18]. What’s more, the conventional PSO algorithm [19] as shown in several studies can easily fly into the local optima. It also lacks the ability to jump out of the local optima when solving complex multimodal tasks. The SOA simulates the act of human searching and has been widely developed for system identification [20]. Nonetheless, the performance of SOA is also affected by its parameters, and it could not easily escape from premature convergence.

Differential evolution, proposed by Storn and Price [21], is a population-based heuristic search algorithm with dual features of reliability and flexibility. It implements the evolutionary generation-and-test paradigm for global optimization by using the current population information of distance and direction to guide the search. It has many advantages such as simplicity, reliability, high performance and easy implementation, which gives great potential application to IIR design. In the seminal DE algorithm, perturbation is operated by adding a weighted moving vector (the weight *F* is called scale factor) and modifying the values of some randomly selected coordinates. The perturbed solution, namely the offspring, is then evaluated by means of its objective function and compared with its corresponding parent. If the newly generated solution outperforms its parent, then a replacement occurs; otherwise the parent solution is retained. To provide a rigorous proof for its probabilistic convergence, [22] has modeled the population as a dynamical system in which the probability density function (PDF) of the population vectors changes with time. It was shown therein that the dynamics is asymptotically stable (which implies convergence) at the equilibrium PDF, which is a Dirac delta function placed at the global optima. Later on, various mutation strategies [23] were used for the generation of new solutions to augment the robustness of the underlying algorithm.

In DE, it is often the case that, for optimization problems such as single-objective, multi-objective, large scale, constrained and dynamic problems, the population size is naturally fixed on a constant value; see, e.g., [24]. Unfortunately, it is usually difficult to determine how large the population size is suitable for solving numerical optimization problems. For instance, a definite population size is given in [25] which increases linearly with the problem dimension; yet the sparse and noisy data makes it difficult to accurately estimate the maximum population size. Inspired by this fact, an efficient population utilization strategy for DE (DESAP) [26] was developed to automatically tune its population size from initialization to completion right through the evolutionary search process. Nevertheless, the population utilization method depends on its encoding methodology, which is a restriction for the current population with complex dynamical behaviors [27]. No significant advantages can be observed while using relative encoding. Subsequently, the idea of population adaptation has been applied in solving dynamic optimization problems [28], where multi-population approach (DynDE) is placed onto DE aimed at locating optima faster. Yet such a method requires a determinated topology that may be sensitive to the noise of measurement in some extent [29]. It is worth mentioning that although the population may not be as large as possible, it ought to meet the requirements of given engineering. Therefore, a new reduction method for the population size was shown up for the jDE in order to enhance algorithmic performance [30], where the population size was progressively declining until the arrival of the final budget during the optimization process. Unfortunately, this method can not keep track of the progress of individuals in the sustainable reduction.

Many studies have indicated that various computational predictors or models developed in biology and biomedicine, such as those in identifying DNA-binding proteins [31], predicting G-protein-coupled receptors (GPCRs) and their types [32], identifying nuclear receptors and their subfamilies [33], [34], identifying the subcellular localization of proteins from various organisms [35]–[39], can timely provide very useful insights and informations for both basic research and drug development. These predictors all use the methods of digital signal processing. In view of this, the present study is attempted to addresses an important problem in designing digital IIR filters in hopes that it may become a useful tool for the related information-treating areas. However, most of the developed adaptive population methods have their advantages and disadvantages. So far, it remains open that how to utilize the dynamic population strategy to solve real-world practical problem. We aim at employing a Markov jumping (switching) population updating DE for digital IIR filter, so that the dynamic population can quickly converge to the potential global optimum by taking advantage of the current search information. Thus, the CPDE-based evolutionary method is simulated in digital IIR filter design, and its performance is compared to that of three versions of DE, CMA-ES, GL-25 and SOA. In the community of six digital IIR filter design problems, it is shown empirically that CPDE is capable of producing highly competitive results compared with other EAs.

## Results

### 0.1 Illustration

**Figure 1:**
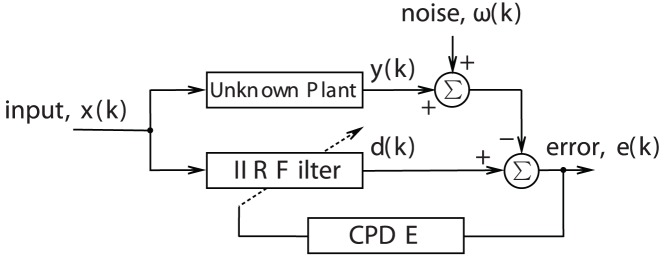
Block diagram of the system identification process using IIR filter designed by CPDE.

Application of the IIR filter in system identification has been widely studied since many problems encountered in signal processing can be characterized as a system identification problem (Figure 1) [40], [41]. Therefore, in the simulation study, IIR filters are designed for the system identification purpose. In this section, we will utilize a modified DE to adjust the parameters of the filters until the error between the output of the filter and the unknown system is minimized. Subsequently, we provide an overall comparison between the performance of CPDE and several other State-of-the-Art algorithms to verify the effectiveness and usefulness of the proposed method.

Six typical system identification problems [14] make up the test suite used for this comparative study, which are listed in Tables 1 and 2. 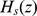 and 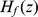 specify the system and filter transfer functions, respectively; 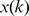 indicates the system input; SNR is the Signal to Noise Ratio; 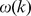 is the system noise, which is independent of 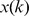; 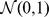 presents the white Gaussian noise (WGN) in zero-mean normal distribution with variance 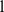. In Table 2, 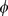 records all coefficients of six digital IIR filters; Search space is the predefined boundary constraints, that will be analyzed in Section 05; 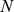 denotes the data length used in calculating the mean-square-error (MSE). The examples were selected so as to include problems with the following characteristics: unimodal/multi-modal, no noise/noisy. For each algorithm and each test function, 30 independent runs are conducted with 100,000 FES as the termination criterion.

**Table 1:** Problem Illustration.

Inst.	Test Function	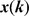	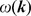
Example 1	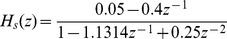	a white-noise sequence	
	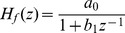		
Example 2	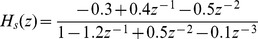	a uniformly distributed white-noise sequence, taking values from 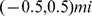 , 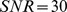 dB	
	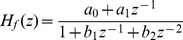		
Example 3	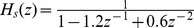	a unit-variance white Gaussian pseudonoise sequence	0and
	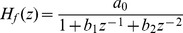		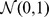
Example 4	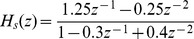	a white-noise input, 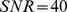 dB	
	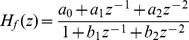		
Example 5	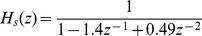	a colored noise by filtering a white Gaussian pseudo-noise sequence with a FIR filter:	
	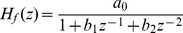	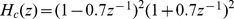	
Example 6	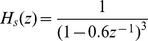	a colored noise by filtering a white Gaussian pseudo-noise sequence with a FIR filter:	
	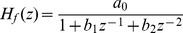	in 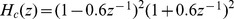	

**Table 2:** Parameters Illustration.

Inst.	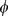	Search Space	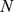
Example 1	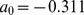		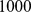
	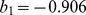		
Example 2	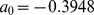 , 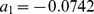		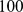
	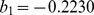 , 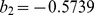		
Example 3	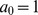		100
	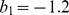 , 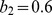	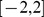	
Example 4	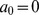 , 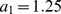 , 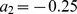		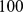
	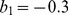 , 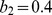		
Example 5	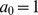		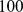
	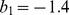 , 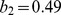		
Example 6	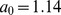		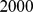
	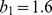 , 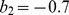		

Traditionally,”generation” is a natural form of computational cost for statistical comparison [11]–[14], [17]. However, the population may not be the same in different algorithms. The algorithm with a larger population may obtain a better performance together with much more function evaluations in every generation. Thus, in this paper, the function evaluations (FES) are conducted here to represent its computational cost for algorithm comparison.

In all simulations, the population size of the most EAs is 100 with the exception of EPSDE and SaDE. As suggested in Ref. [42], [43], the population size of EPSDE and SaDE is chosen to be 50. Seven existing EA algorithms are shown in Table 3 in detail. CMA-ES represents the state of the art of Evolution Strategies and it is a referent in the continuous optimization field. GL-25 is a hybrid real-coded genetic algorithm which combines the global and local search. EPSDE is an adaptive DE with ensemble of parameters which incorporates a self-organizing method. jDE is a standard DE with adapted parameter setting. SaDE delivers a mutation strategy pool where strategy is self-adapted based on their previous performance. SOA is a novel heuristic stochastic optimization algorithm based on the simulation of the act of human searching. The parameters for these EAs are provided in Table 3.

**Table 3:** EA algorithms for comparison.

Algorithm	Parameters	Reference
CMA-ES¡¡	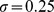	[54]
GL-25¡¡	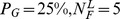	[55]
EPSDE¡¡	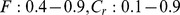	[42]
jDE¡¡	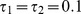	[56]
SaDE¡¡	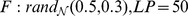	[43]
SOA	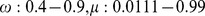	[14]
CPDE	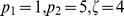	this paper

### 0.2 Comparison on the Solution Accuracy

**Figure 2:**
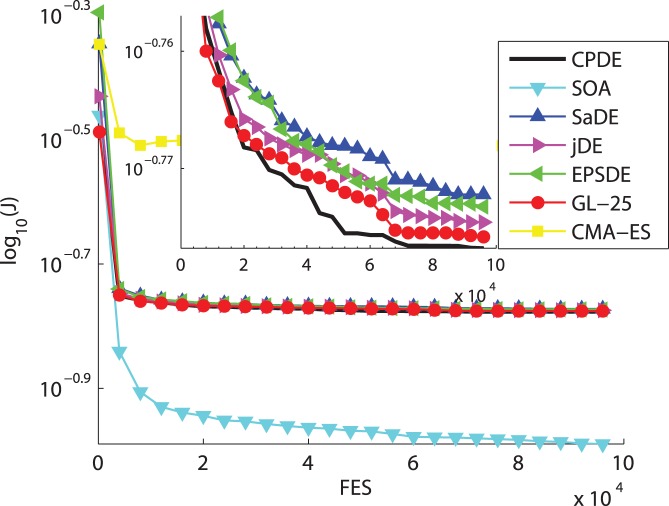
Cost function value versus number of evaluations averaged over 30 random runs for the seven algorithms (a) 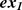
 **. (b)** 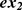 **. (c)** 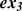 **. (d)** 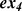 **. (e)** 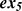 **. (f)** 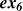 **.**

In this section, an overall comparison of the performance is provided between the CPDE variant and other six State-of-the-Art EAs (i.e., CMA-ES, GL-25, EPSDE, jDE, SaDE and SOA). We evaluate the performance of seven heuristic algorithms over six typical nonlinear uncertain discrete-time problems. Fig. 2 illustrates the cost function value versus number of evaluations averaged over 30 random runs for the seven algorithms. The subfigures amplify the convergence graphs in clarity. Table 4 reports the experimental results of Examples 1–6, averaged over 30 independent runs with 100,000 FES.

**Table 4:** Experimental results of Examples 1–6, averaged over 30 independent runs with 100,000 FES.

Inst.		CMA-ES	GL-25	EPSDE	jDE	SaDE	SOA	CPDE
	Mean Error	3.2236E–01	1.6756E–01	1.6856E–01	1.6804E–01	1.6896E–01	**1.0246E–01**	1.6719E–01
Example 1	Std Dev	2.1399E–01	2.2370E–03	2.9506E–03	2.3747E–03	2.2793E–03	**4.2071E–03**	2.1508E–03
	T-test	+	+	+	+	+	–	
	Mean Error	1.4424E–02	6.8069E–03	6.7232E–03	6.6451E–03	6.9145E–03	6.6265E–03	**6.3136E–03**
Example 2	Std Dev	5.9387E–03	4.4369E–04	4.4507E–04	4.1499E–04	4.7206E–04	3.1431E–04	**5.0418E–04**
	T-test	+	+	+	+	+	+	
	Mean Error	1.4517E–01	8.9999E–33	0.00E+00	0.00E+00	0.00E+00	0.00E+00	**0.00E+00**
Example 3	Std Dev	5.1851E–01	3.4254E–32	0.00E+00	0.00E+00	0.00E+00	0.00E+00	**0.00E+00**
	T-test	+	+	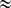	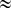	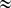	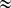	
	Mean Error	1.2372E–04	4.6732E–64	1.4751E–71	4.2794E–73	1.4999E–83	3.9896E–100	**0.00E+00**
Example 4	Std Dev	6.7762E–04	2.5596E–63	4.5215E–72	2.0951E–73	6.7257E–83	1.8653E–99	**0.00E+00**
	T-test	+	+	+	+	+	+	
	Mean Error	2.4285E–01	3.9674E–20	0.00E+00	0.00E+00	0.00E+00	0.00E+00	**0.00E+00**
Example 5	Std Dev	6.0577E–01	1.8016E–19	0.00E+00	0.00E+00	0.00E+00	0.00E+00	**0.00E+00**
	T-test	+	+	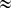	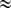	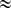	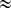	
	Mean Error	1.8801E+00	1.0167E–01	1.0162E–01	1.0127E–01	1.0199E–01	1.0168E–01	**1.0063E-01**
Example 6	Std Dev	2.9858E+00	1.3400E–03	1.6198E–03	9.8318E–01	1.3437E–03	1.2518E–03	**1.1027E–03**
	T-test	+	+	+	+	+	+	

**Figure 3:**
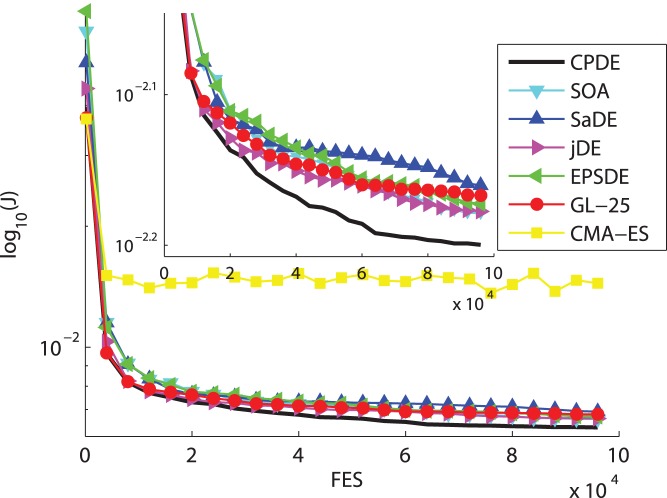
Instance of evolution of the parameters of two filters for CPDE (a) 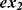
 **. (b)** 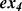 **.**

From the Table 4 and Fig. 2, the CPDE provides the best performance on the 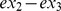, 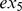 and 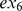, then ranks the second on the 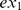 and 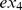. SOA gives the best performance on the 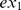 and 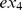. The results show that GL-25 and SOA have good ability of convergence speed. Fig. 3 also shows instance of evolution of the parameters of two filters for CPDE.

To be specific, on the three multimodal problems (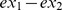 and 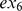), CPDE performs much better than CMA-ES, GL-25, EPSDE, jDE, SaDE and SOA on the latter two functions. SOA delivers good accuracy and the highest convergence rate on 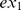, while CPDE outperforms other five methods. To sum up, CPDE is the winner on multimodal functions. This might be due to the fact that CPDE implements the overall adaptive variable population size method, which can help the DE search the optimum as well as maintain a higher convergence speed when dealing with multimodal rotated functions. On the remaining three unimodal functions (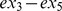), CPDE performs significantly better than six others for 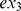 and 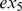. SOA can provide good accuracy on 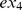, while CPDE achieves the highest convergence rate. The outstanding performance of CPDE is due to its dynamic PDS, which leads to very fast convergence. Overall, the CPDE is the best among the seven methods in the comparison conducted on unimodal functions and expanded multi-modal functions. For a thorough comparison, the t-test has also been carried out in this paper. Table 4 presents the total score on every function of this two-tailed test with a significance level of 0.05 between the CPDE variant and other heuristic algorithms. Rows “+ (Better),” “ =  (Same),” and “– (Worse)” give the number of functions that the CPDE performs significantly better than, almost the same as, and significantly worse than the compared algorithm on fitness values in 30 runs, respectively. As confirmed in t-test, the CPDE in general offers more improved performance than the other six State-of-the-Art EAs.

As mentioned above, the CPDE has shown a very competitive performance in the six filtering problems. In practical engineering, noise exist universally in nature [44]. Therefore, in the past few decades researchers have witnessed significant progress on filtering and control for linear/nonlinear systems with various types of noises among which Gaussian noise is one of the most general signals that has been widely studied [45], [46]. Here, we further evaluate the proposed framework on the six expanded stochastic systems, where a zero-mean Gaussian white-noise is added. The maximum number of FES is set to be 100,000 in all runs. Table 5 summarizes the experimental results.

**Table 5:** Experimental results of Examples 1–6 with noise perturbation, averaged over 30 independent runs with 100,000 FES.

Inst.		CMA-ES	GL-25	EPSDE	jDE	SaDE	SOA	CPDE
	Mean Error	1.3351E+00	9.9644E–01	1.0013E+00	9.9526E–01	1.0001E+00	**6.4329E–01**	9.8494E–01
Example 1	Std Dev	2.2756E–02	1.3799E–02	1.1911E–02	1.3331E–02	1.5337E–02	**2.7742E–02**	1.2588E–02
	T-test	+	+	+	+	+	–	
	Mean Error	8.6191E–01	5.2458E–01	5.5239E–01	5.3305E–01	5.4549E–01	5.4789E–01	**5.1608E–01**
Example 2	Std Dev	7.7377E–02	2.7869E–02	1.9928E–02	2.3498E–02	2.2388E–02	2.2444E–02	**1.8810E–02**
	T-test	+	+	+	+	+	+	
	Mean Error	1.0701E+00	5.3043E–01	5.3999E–01	5.3420E–01	5.4608E–01	5.4132E–01	**5.0512E–01**
Example 3	Std Dev	6.8551E–01	2.2359E–02	2.9233E–02	2.3386E–02	2.5721E–01	2.2572E–02	**2.2874E–02**
	T-test	+	+	+	+	+	+	
	Mean Error	8.9973E–01	5.4311E–01	5.5414E–01	5.3367E–01	5.5998E–01	5.3624E–01	**5.2156E–01**
Example 4	Std Dev	7.9829E–02	2.8612E–02	2.5562E–02	2.1445E–02	3.2593E–02	2.6400E–02	**2.1444E–02**
	T-test	+	+	+	+	+	+	
	Mean Error	1.6026E+00	5.3366E−01	5.4711E–01	5.2517E–01	5.4717E–01	5.2705E–01	**5.0945E–01**
Example 5	Std Dev	1.1095E+00	2.8589E–02	2.7466E–02	2.5575E–02	2.4434E–02	3.4826E–02	**1.6459E–02**
	T-test	+	+	+	+	+	+	+
	Mean Error	2.7866E+00	9.8570E–01	9.8841E–01	9.8231E–01	9.8739E–01	9.8765E–01	**9.7624E–01**
Example 6	Std Dev	2.9110E+00	1.3256E–02	9.7794E–03	9.2576E–03	1.3060E–02	1.0416E–02	**7.8179E–02**
	T-test	+	+	+	+	+	+	

From the Table 5, the CPDE provides the best performance on the 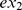, 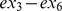, then ranks the second on the 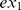. SOA offers the best performance on the 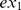. The results show that GL-25 and jDE have a good ability of convergence speed.

More specifically, on the three multimodal problems (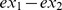 and 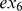), although it worked slightly weaker on some functions, the CPDE in general offered more improved performance than all the EAs compared. It performs much better on the 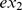 and 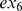, and attains slightly worse performances than the best solutions on the 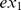. On the remaining three unimodal functions (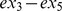), CPDE performs much better than other EAs. Hence, CPDE exhibits the highest performance in noise-expanded filtering problems, which can efficiently adjust the population structure and guide the evolution process toward more promising solutions.

The t-test is also summarized in Table 5. In fact, CPDE performs better than CMA-ES, GL-25, EPSDE, jDE, SaDE and SOA on 6, 6, 6, 6, 6 and 5 out of 6 test functions, respectively. Thus, CPDE is better than other six competitors in filtering system identification problems. Comparing the results and the cost function graphs, among these seven EA algorithms, the GL-25 can converge to the best solution found so far very quickly though it is easy to stuck in the local optima. The SOA has good global search ability and slow convergence speed. The jDE have good search capability on noise-expanded filtering problems. The CPDE has good local search ability and global search ability at the same time.

### 0.3 Comparison on Convergence Rate and Successful Percentage

The convergence rate for achieving the global optimum is another key point for testing the algorithm performance. Note that in solving real-world optimization problems, the “function evaluation” overwhelms the algorithm overhead [47]. Hence, the computation times of these algorithms are not provided here. Table 6 shows that CPDE needs the least FES to achieve the acceptable solution on 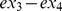 and 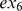, which reveals that CPDE has a higher convergence rate than other algorithms. Though SOA or GL-25 might outperform CPDE on the other functions, SOA and GL-25 have much worse successful ratio and accuracy than CPDE on the problems for comparison. In addition, CPDE can achieve accepted value with a good convergence speed and accuracy on most of the problems, as shown in Tables 4, 5 and Fig. 2. Tables 4, 5 also show that CPDE yields a highest percentage for achieving acceptable solutions in 30 runs. According to the no free lunch theorem [48], any elevated performance over one class of problems is offset by performance over another class. Hence, one algorithm cannot perform better on convergence speed and accuracy than the others on every optimization problem.

**Table 6:** Convergence speed and algorithm reliability comparisons on Examples 1–6 with noise perturbation; ‘
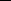
’ representing no runs reached an acceptable solution.

Inst.		CMA-ES	GL-25	EPSDE	jDE	SaDE	SOA	CPDE
Example 1	Mean Generations	–	313.5	438.6	397.1	380.5	**10.8**	341.7
	Right Percentage(%)	0	86.7	73.3	90	76.7	**100**	**100**
Example 2	Mean Generations	406.7	**295.9**	378.6	370.3	477.3	587.9	356.8
	Right Percentage(%)	56.7	90	70	86.7	73.3	56.7	**96.7**
Example 3	Mean Generations	–	554.1	560.6	454.6	513.9	716.8	**406.5**
	Right Percentage(%)	0	66.7	53.3	66.7	36.7	63.3	**100**
Example 4	Mean Generations	329.2	313.2	467.5	493	508.5	546.6	**290.4**
	Right Percentage(%)	23.3	90	73.3	90	66.7	90	**100**
Example 5	Mean Generations	–	388.1	422.5	**295.4**	516.6	513.1	363.3
	Right Percentage(%)	0	90	80	93.3	66.7	**100**	**100**
Example 6	Mean Generations	–	340.3	510	298.7	364.8	520.4	**291.9**
	Right Percentage(%)	0	90	80	93.3	86.7	**100**	**100**
	Mean Reliability	13.3	85.6	71.6	86.7	67.8	85	**99.45**

In summary, the CPDE performs best on unimodal problems with or without noise and has a good search ability of multimodal problems. Owing to the controllable probabilistic technique, the CPDE processes capabilities of fast convergence speed, highest successful ratio and the best search accuracy among these EAs.

### 0.4 Performance of Controllable Probabilistic Approach

In this section, the controllable probabilistic (CP) approach is used to test the search performance of CPDE. In all the experiments, threshold 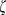 is adjusted in the following. Moreover, the parameter 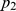 is also considered, which denotes the number of potential candidates for perturbation.

In this paper, 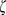 indicates the trigger thresholds, which is used to control the sensitivity of the dynamic CPDE. While 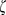 is set as one, the population size will be adjusted in each generation. Setting a higher 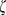 value will result in a lower sensitivity of the CPDE, while a lower 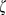 value will lead to a higher efficiency of the population adjustment. Notice that the parameter 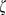 should set to be larger than one. Failure to do this will result in an instant elimination of a newborn individual with poor performance, which may provide some degree of diversity preservation. On the other hand, coefficient 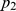 also influences the perturbation process substantially.

Table 7 shows the comparisons between CPDE with other three parameter settings of CPDE over Examples 1–6 with noise perturbation. It indicates that CPDE is not sensitive to the adjustment of parameters. In order to make a balance of the search accuracy and robustness, 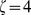 and 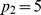 are used as a representative parameter setting in our paper. This setting will prevent the instant elimination of a newborn individual and keep the CP approach high sensitivity.

**Table 7:** Effects of 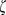 and 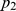 on search accuracy of CPDE.

Inst.	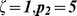	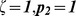	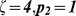	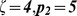
	Mean Error ± Std Dev	Mean Error ± Std Dev	Mean Error ± Std Dev	Mean Error ± Std Dev
Example 1	9.8904E–011.2771E–02≈	9.8780E–011.1698E–02	9.9147E–01±7.5527E–03+	9.8494E–01±1.2588E–02
Example 2	5.1568E–01±2.8237E–02	5.3038E–01±1.7731E–02+	5.2603E–01±2.1477E–02+	5.1608E–01±1.8810E–02
Example 3	5.1935E–01±1.6915E–02+	5.0609E–01±2.3654E–02	5.2400E–01±2.2003E–02+	5.0512E–01±2.2874E–02
Example 4	5.2926E–01±2.5568E–02	5.2539E–01±2.5586E–02≈	5.2821E–01±2.5189E–02≈	5.2156E–01±2.1444E–02
Example 5	5.1930E–01±2.5434E–02+	5.1788E–01±2.3220E–02+	5.2145E–01±3.0849E–02+	5.0945E–01±1.6459E–02
Example 6	9.7708E–01±1.2567E–02≈	9.7858E–01±1.1414E–02≈	9.7989E–01±8.3052E–03≈	9.7624E–01±7.8179E–02

## Discussion

The CPDE is an improved differential evolution algorithm with a controllable probabilistic population size. When particles are clustered together in a region and trapped into the local basin, CPDE perturbs the population and generates the necessary “fine” individuals to share their up-to-date information. In addition, CPDE removes redundant individual with its entropy and ranking metrics to save computational load. In this paper, a CPDE-based digital filter design method has been proposed, and the benefits of CPDE for designing digital IIR filters have been studied.

An overall comparison between the performance of the CPDE and other six State-of-the-Art EAs (i.e., jDE, SaDE, EPSDE, CMA-ES, GL-25 and SOA) was provided over 6 typical robust system identification problems, and the result clearly indicated the CPDE achieved a substantially significant improvement on the performance. Furthermore, convergence rate was also validated that the CPDE has good convergence performance to achieve the fixed accuracy level with acceptable generations. Thus, it is believed that the proposed CPDE is capable of rapidly and efficiently adapting the parameters of a wide variety of IIR structures and will become a promising candidate for digital filter design.

Previous work has shown the importance of system identification on digital IIR filter design. Furthermore, CPDE has effectively been applied to estimate the structure of nonlinear uncertain discrete-time system. Therefore, our method is possible to be used to reconstruct the topology structure for on-line adaptive filtering applications [49]. Another possible application is to identify topology and parameters of complex networks [50]–[52] and biological time series [53] by dynamic population strategy, provided online measurement and increasing/decreasing techniques are feasible. Generally, the suggested technique enables us to identify the unknown parameter of real networks which allows the required control applications (perturbations). Some possible experimental research is now under our investigation in controller design and tuning.

## Materials and Methods

### 0.5 Description of the Problem

Consider the digital IIR filter with the input-output relationship governed by the difference equation:
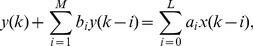
(1)where 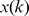 and 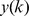 are the filter’s input and output, respectively, and 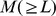 is the filter order. The transfer function of this IIR filter can be written in the following general form:



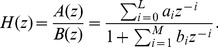
(2)Hence, the design of this filter can be formulated as an optimization problem with the cost function 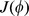 stated as follows:
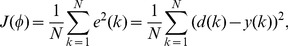
(3)where 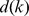 and 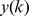 are the desired and actual responses of the filter, respectively; and 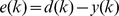 is the filter’s error signal; 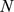 is the number of samples used to calculate the objective function.
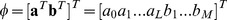
(4)denotes the filter coefficient vector. The aim is to minimize the cost function 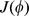 by adjusting 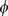. An important consideration during the adaptive process is to maintain the stability of the IIR filter. Not all filters defined by Eq. (1) are feasible or implementable. An efficient way of maintaining stability is to convert the direct form to a lattice form and make sure that all reflection coefficients 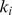, 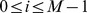, have magnitudes less than one. We will adopt a similar approach as in [11] to guarantee the stability of the IIR filter during adaptation. Thus, the actual filter coefficient vector used in optimization is 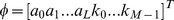. In the circumstances, the coefficient space 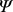 is formed by the constraints of 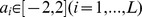 and the magnitudes of 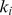 that are less than one. For the sake of simplicity, we adopt the predefined boundary constraints as 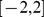 to compare other existing EAs fairly.

### 0.6 A Controllable Probabilistic DE

The stochastic system is iterated forward in time using a synchronous DE updating scheme. In our work, a mode-dependent population updating equation with Markovian switching parameters is introduced with the hope to keep track of the progress of individuals and further improve the search abilities. A detailed algorithm design of CPDE can be found in **Documentation S1**.

In CPDE, there are two levels of sub-optimizers, population decreasing strategy (PDS) and population increasing strategy (PIS). The probability of selecting different sub-optimizer to improve the online solution-searching status is completely up to its non-homogeneous Markov chain. For choosing required sub-optimizers adaptively, consider the following probability transition matrix in Eq. (5):
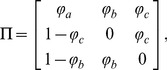
(5)


Here, 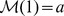, 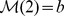 and 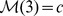 stand for the population maintaining state, population increasing state and population decreasing state, respectively. The expectations of Markov chain 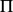 are automatically updated by the search environment. It is worthwhile to mention that 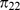 and 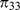 are set to be 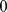. In this case, the increasing and decreasing operators will not be performed in consecutive generations.

If few trial vectors can outperform the corresponding parent in selection operation, particles may be clustered together and trapped into the local basin. In such a case, the PIS is employed to add new individuals into the population and share their up-to-date information to help the individuals escape the local basin.
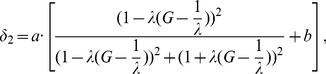
(6)where 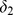 is the number of dimensions for perturbation, which is monotonically decreasing through the evolutionary search. Parameters *a* and *b* are the magnification coefficient. Parameters 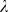 and 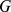 are here considered as the generation variables. During early stages of the optimization process, much more reproductions will be generated to spread out its particles within the decision space. Nevertheless, solutions of the population tend to concentrate in specific parts of the decision space during the later period of optimization process.

However, in case most of individuals can spawn new promising offspings in the evolutionary process, it then signifies that redundant intermediate particles exist. In this case, we introduce the PDS to remove poor particles to avoid undesirable computational cost and excessive search complexity.
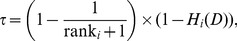
(7)where 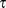 denots an overall deletion indicator. The variable 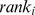 and 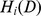 indicate the rank metric and the entropy metric for individual 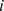, respectively. It can be observed that from Eq. (7), for the individuals that have high rank values (i.e., away from the global best solution) or low entropy values (i.e., located in the crowded regions); these particles will have a higher probability of elimination.

## Supporting Information

Documentation S1 **A material algorithm design of CPDE.**
(PDF)
