# Supplementary material for: Digital IIR Filters Design Using Differential Evolution Algorithm with a Controllable Probabilistic Population Size
Source: PLoS One. 2012 Jul 11;7(7):e40549. doi: 10.1371/journal.pone.0040549 (PMC3394744; doi:10.1371/journal.pone.0040549)
Supplement: Documentation S1 — A material algorithm design of CPDE. (PDF) [file pone.0040549.s001.pdf]

Documentation S1

A material design of CPDE

Algorithm design

In this paper, we aim at employing an improved DE for digital IIR filter design. The CPDE-based evolutionary method is elaborated in [1]. For the sake of simplicity, we just repeat the details in [1] here. It is a mode-dependent population updating strategy with Markovian switching parameters with the hope to keep track of the progress of individuals and further improve the search abilities, which has been briefly introduced as follows.

In general, a *population increasing strategy* (PIS) and a *population decreasing strategy* (PDS) are applied to update the particles, as the swarm population progresses at each iteration. Furthermore, the tactic also adjust population when it stagnates at the lower or upper bound. For choosing different strategies adaptively, consider the following probability transition matrix in Eq. (1):

$$\Gamma = \begin{bmatrix} \varphi_a & \varphi_b & \varphi_c \\ 1-\varphi_c & 0 & \varphi_c \\ 1-\varphi_b & \varphi_b & 0 \end{bmatrix}, \tag{1}$$

where π_{ij} denotes the transition rate from i to j and $\sum_{j=1}^{3} \pi_{ij} = 1$. Here, $\mathcal{M}(1) = a$, $\mathcal{M}(2) = b$ and $\mathcal{M}(3) = c$ stand for the population maintaining state, population increasing state and population decreasing state, respectively. It is worthwhile to mention that π_{22} and π_{33} are set to be 0. At each generation G, the conditional probability φ_b and φ_c is independently generated according to a Cauchy distribution with location parameters $boot_b$ and $boot_c$, as well as standard deviation 0.1.

$$\varphi_b = Cauchy(boot_b, 0.1), \tag{2}$$

$$\varphi_c = Cauchy(boot_c, 0.1), \tag{3}$$

$$\varphi_a = 1 - \varphi_b - \varphi_c, \tag{4}$$

and then truncated to be 1 if $\varphi_b + \varphi_c > 1$ or regenerated if $\varphi \leq 0$. Denote σ_b as the proportion distribution of trial vectors that are discarded in the next generation, while σ_c as the proportion distribution of trial vectors that can successfully enter the next generation. As illustrated in Eq. (7), the numbers of successful and unsuccessful trial individuals are recorded as $n_{s,G}$ and $n_{f,G}$, respectively. Meanwhile, the location parameter *boot* of the Cauchy distribution is initialized to be 0.1 and then updated at the end of each generation as Eqs. (5)-(6):

$$boot_b = (1-k) \cdot boot_b + k \cdot \mathcal{F}(\sigma_b), \tag{5}$$

$$boot_c = (1-k) \cdot boot_c + k \cdot \mathcal{F}(\sigma_c), \tag{6}$$

$$\sigma_b = 1 - \sigma_c = \frac{n_{f,G}}{n_{s,G} + n_{f,G}}, \tag{7}$$

where k is a positive constant between 0 and 1, and it controls the rate of parameter adaptation. Similar to Ref. [2], the *life span* of a successful φ_b or φ_c is roughly $1/k$ generations; i.e., after $1/k$ generations, the old value of $boot_b$ or $boot_c$ is reduced by a factor of $(1-k)^{1/k} \longrightarrow 1/e \approx 37\%$, when k is close to zero. Hence, we consider the life span of φ_b or φ_c values 10 generations and set the parameter $k = 0.1$ in all simulations for fair comparison.

In addition, a normalization operator is employed to transform σ into an exponential distribution so that each generation step has a different value. This novel transformation of each proportion distribution σ is assigned as Eq. (8):

$$\mathcal{F}(\sigma) = \mathcal{F}_1 + (\mathcal{F}_2 - \mathcal{F}_1) * \frac{e^{4\sigma} - 1}{e^4 - 1}, \tag{8}$$

Algorithm 1 Controllable Probabilistic Differential Evolution

```
Begin
    i = 0, flag = 1
    Create a population of PS vectors randomly with PS ∈ [Lbound, Ubound]
    while The halting criterion is not satisfied do
        Procedure of DE algorithm
        i = i + 1
        if i == \zeta then
            Compute \varphi_a, \varphi_b and \varphi_c according to Eqs. (2)-(4)
            \mathcal{R} = rand[0, 1]
            switch flag
                case 1
                    if \mathcal{R} \leq \varphi_a then
                        flag = 1
                    else if \mathcal{R} \leq \varphi_a + \varphi_b then
                        if PS < Ubound then
                            Increasing-Strategy()
                        else
                            Increasing-Strategy()
                            Decreasing-Strategy()
                        end if
                        flag = 2
                    else
                        if PS > Lbound then
                            Decreasing-Strategy()
                        end if
                        flag = 3
                    end if
                case 2
                    if \mathcal{R} \leq \varphi_a then
                        flag = 1
                    else
                        if PS > Lbound then
                            Decreasing-Strategy()
                        end if
                        flag = 3
                    end if
                case 3
                    if \mathcal{R} \leq \varphi_a then
                        flag = 1
                    else
                        if PS < Ubound then
                            Increasing-Strategy()
                        else
                            Increasing-Strategy()
                            Decreasing-Strategy()
                        end if
                        flag = 2
                    end if
            End
            i = 0
        end if
    end while
End
```

where $\mathcal{F}_2$ and $\mathcal{F}_1$ are the upper and lower bounds of $\mathcal{F}(\sigma)$. We set $\mathcal{F}_1 = 0.05$ and $\mathcal{F}_2 = 0.5$ [3] in this paper, which indicates that $\mathcal{M}(2)$ occurs frequently and $\mathcal{M}(3)$ seldom happens in a stage of stagnation. Conversely, in the period of progress, $\mathcal{M}(2)$ seldom occurs and $\mathcal{M}(3)$ happens frequently, which refines the solution efficiently. This mechanism can make the swarm benefit from the global search as well as local search.

As elaborated in **Algorithm 1**, *Ubound* and *Lbound* are upper and lower bounds of the population size, respectively. On this occasion, the population dynamic state, $\mathcal{M}(2)$ or $\mathcal{M}(3)$, can not be continuously repeated in 4 consecutive generations by reason of $\pi_{22} = \pi_{33} = 0$ in Eq. (1). Besides, population adjustment is also adopted when it stagnates at the upper bound.

0.0.1 Population Increasing Strategy

• Scale of Perturbation

For CPDE with dynamic population size, the superior of individuals selected for reproduction is also dynamic so that the desired diversity of perturbation could be generated. The proposed potential candidates are calculated upon Eq. (9):

$$\delta_1 = \lceil\ p_2\% \times PS\ \rceil, \tag{9}$$

where function $\lceil a \rceil$ represents the smallest integer that is greater than or equal to a. δ_1 is the number of individuals for reproduction. Once δ_1 is determined, the chosen potential candidates are stored into a temporary memory, called Elite-Archive (EA). EA should be emptied at the end of every generation.

• Location of Perturbation

Perturbation operation is performed on each potential candidate in EA. A smooth continuous function can be used to ensure that the number δ_2 for perturbation of the selected particle is adaptively time-varying. The number δ_2 is computed upon Eq. (10):

$$\delta_2 = a \cdot [\frac{(1-\lambda(G-\frac{1}{\lambda}))^2}{(1-\lambda(G-\frac{1}{\lambda}))^2 + (1+\lambda(G-\frac{1}{\lambda}))^2} + b], \tag{10}$$

$$\lambda = \frac{2}{G_{max}}, \qquad 0 \leq G \leq \frac{2}{\lambda} \tag{11}$$

$$a = U_\delta - L_\delta, \qquad b = \frac{L_\delta}{U_\delta - L_\delta}, \tag{12}$$

where a and b denote the magnification coefficient, which are generated with Eq. (12). U_δ and L_δ are the maximum and the minimum possible value of δ_2. For simplicity, they are set in this paper as 5 and 1 [4], respectively. G_{max} indicates the maximum generation.

• Distance of Perturbation

Following the idea of [5], an incremental distance is defined to perturb the selected candidate in δ_2 dimensions. Thus, a normal distribution is adopted to tune the additional distance and to avoid children

being too far away from their parents. The incremental distance is calculated upon Eq. (14):

$$R = abs(Gaussian(0, 1/9)), \tag{13}$$

$$\Delta d(R) = a \cdot [\frac{(1+\lambda(R-\frac{1}{\lambda}))^2}{(1-\lambda(R-\frac{1}{\lambda}))^2 + (1+\lambda(R-\frac{1}{\lambda}))^2} + b], \tag{14}$$

$$\lambda = 2, \qquad 0 \leq R \leq 1 \tag{15}$$

$$a = U_d - l_d, \qquad b = \frac{l_d}{U_d - l_d}, \tag{16}$$

$$U_d = c_1 \times x_j^U, \qquad l_d = c_2 \times x_j^l, \tag{17}$$

$$x_i^j = x_i^j + \Delta d(R), \tag{18}$$

where R denotes the intermediate parameter, which is approximated by a normal distribution with mean value 0 and standard deviation 1/9. U_d and l_d are the upper and lower limits respectively of the incremental distance, which are calculated by Eq. (17), where x_j^U and x_j^l are the upper and lower boundaries for the decision variable x in dimension j, and c_1, c_2 are the user-defined scaling ratios. For simplicity, the coefficients c_1 and c_2 are chosen in the range of $0.02 \leq c_1 \leq 0.7$ and $0 \leq c_2 \leq 0.02$, which are determined based on the desired boundary of the incremental distance. As mentioned above, with the mean 0 and variance 1/9, the parameter R is more likely located in the range of $[0, 0.5]$, and therefore the probability of perturbation is higher within the "neighborhood region" than outside.

It should be noted that the incremental individuals may violate predefined boundary constraints. In this case, resetting scheme can be used to tackle this problem. When the perturbation $x_{i,G}^j + \Delta d$ is infeasible (i.e., out of the boundary), the counter j will be decreased by unity, and the perturbation on the jth dimension wil be reperformed instantly.

0.0.2 Population Decreasing Strategy

• **Entropy Metric**

The entropy value has been used as a measure of diversity in evolutionary algorithms, providing information regarding the spread of the individuals' fitness values. Thus, monitoring its value during the optimization procedure provides information regarding the population's behavior. To describe this approach, we use a distance-estimation operator, which has been stated in Eqs. (19)-(20):

$$Du(i) = \mid f_{i+1} - f_i \mid, \tag{19}$$

$$Dl(i) = \mid f_i - f_{i-1} \mid, \tag{20}$$

where $Du(i)$ and $Dl(i)$ denote the distances of the i-th particle to its upper and lower adjacent particle along the objective function respectively. Also, the distribution entropy of the i-th solution is defined in Eq. (23):

$$P_{i1} = \frac{Du(i)}{Du(i) + Dl(i)}, \tag{21}$$

$$P_{i2} = \frac{Dl(i)}{Du(i) + Dl(i)}, \tag{22}$$

$$H_i(D) = -\sum_{j=1}^{2} P_{ij} \log_2(P_{ij}), \tag{23}$$

where $H_i(D)$ specifies the overall crowding entropy metric of particle i, which is calculated as the sum of individual crowding entropy values corresponding to either side.

It should be noted that the boundary solutions (solutions with smallest and largest function values) are assigned an infinite crowding entropy value so that which are always selected. All other intermediate solutions are assigned a crowding entropy value according to Eq. (23).

• Rank Metric

This metric involves the notion that the individuals which have high rank values will have less chance to remain in the next iteration because they have a higher probability of losing their leaders. First, the initial objective space should be divided into ω intervals. In each interval, fitness function values will be extensively substituted by an integer pointer, which can be used to determine the performance of each particle, together with generation number. Hence, the interval length can be calculated according to Eq. (24):

$$L = \frac{f_{max} - f_{min}}{\omega}, \tag{24}$$

where f_{max} and f_{min} are the maximum and minimum fitness function values among f_i. ω denotes the trellis scale, which is chosen based upon the interactive knowledge heuristically. In this paper, ω is computed upon Eq. (25):

$$\omega = \lfloor \Omega * popsize \rfloor, \tag{25}$$

where function $\lfloor a \rfloor$ represents the largest integer that is less than or equal to a. The value of Ω should not be smaller than a problem-dependent threshold value 0.5 in order to prevent excessive sparse discrimination, and that if Ω is larger than 1.0, the effectiveness of the ranking method will decrease. So, we suggest that a good initial choice of Ω is 0.8. Then the integer pointer of the i-th particle can be calculated upon Eq. (26):

$$\text{rank}_i = \text{mov}(f_i - f_{min}, L) \ \in [0, \omega]. \tag{26}$$

The function mov(x, y) represents the quotient (integer part) after division x/y. The dividend $f_i - f_{min}$ specifies the distance between particle i and the lower boundary of the current objective space. As a result, the integer pointer $\text{rank}_i \in [0, \omega]$ shows the trellis location of the ith particle in objective space. The particle in a interval with a higher rank value indicates a worse performance, while the particle in a interval with a lower rank value means a better performance.

As the boundary of the range of the objective space and the population size in the decision space will change with different generations, the number and size of intervals will vary from generation to generation to maintain the accuracy of the ranking calculation.

• Diversity Preservation

Following the ranking stage, a normalization operator is employed to sift potential individuals to be deleted. Such a mechanism would, in effect, transform the distribution of rank value from $[0, \omega]$ into $[0, 1]$ as well. Furthermore, this normalization, coupled with a monotone decreasing entropy function, suggests an overall deletion indicator, τ. By the above method, it can be observed that from Eq. (27), for the individuals that have high rank values (i.e., away from the global best solution) or low entropy values (i.e., located in the crowded regions), these particles will have a higher probability of elimination.

$$\tau = (1 - \frac{1}{\text{rank}_i + 1}) \times (1 - H_i(D)). \tag{27}$$

After that, diversity enhancement is performed on each particle whenever a randomly generated number between 0 and 1 is less than its associated τ value. In the circumstances, individual is stored into an Inferior-Archive (IA) and chosen as a potential candidate to be removed from the current population.

IA represents an interim memory, and should be cleared at the end of every generation. Subsequently, in order to determine the specific list of the removal of individuals, a stochastic selection strategy is introduced in Eq. (28):

$$\mathbb{D} = \min\{[\ p_1\% \times PS\ ],\ S_{IA}\}, \tag{28}$$

where $\mathbb{D}$ is the number of individuals for elimination, and S_{IA} is the size of the IA. We randomly select $\mathbb{D}$ worst individuals from IA, and then delete the chosen individuals from the current population. Here, we fix p_1 on a small value, i.e., $p_1 = 1$. Large values of p_1 may result in the indisciplinable removal of a large number of individuals while providing some degree of diversity preservation. Hence, large values of p_1 should always be avoided. Meanwhile, with a small p_1, there has been a tradeoff between the population diversity and the search speed.

Since the elimination in PS is executed to slim the current population, the deleted individuals are transferred into a *grand pool*, named $\mathcal{J}$. In the following mutation, the combination $PS \times \mathcal{J}$ will be used to provide additional information about the promising progress direction. As a demonstration, we applied this operator to the "current-to-rand" strategy, which can be defined as Eq. (29):

$$\mathbf{v}_{i,G} = \mathbf{x}_{i,G} + F_i \cdot (\mathbf{x}_{r1,G} - \mathbf{x}_{i,G}) + F_i \cdot (\mathbf{x}_{r_2,G} - \tilde{\mathbf{x}}_{r_3,G}). \tag{29}$$

Similar to [2], $\tilde{\mathrm{x}}_{r_3,G}$ is randomly selected from the union, $PS \cup \mathcal{J}$, while $\mathcal{J}$, the pool, is employed to store the formerly deleted inferior solutions. This grand pool is initiated to be empty. Then, the addition of the $\mathbb{D}$ particles to the grand pool marks the end of each PDS iteration. If the pool size exceeds a certain threshold, say PS, then some individuals are randomly removed from the pool to keep the pool size at PS.

References

1. Zhu W, Fang J, Tang Y, Zhang WB (2012) Enhanced differential evolution with entropy-based population adaptation and markov chain model. Soft Computing : under review.

2. Zhang JQ, Sanderson AC (2009) Jade: adaptive differential evolution with optional external archive. IEEE Transactions on Evolutionary Computation 13: 945-958.

3. Liang JJ, Qin AK, Suganthan PN, Baskar S (2006) Comprehensive learning particle swarm optimizer for global optimization of multimodal functions. IEEE Transactions on Evolutionary Computation 10: 281-295.

4. Tan KC, Lee TH, Khor EF (2001) Evolutionary algorithms with dynamic population size and local exploration for multiobjective optimization. IEEE Transactions on Evolutionary Computation 5: 565-588.

5. Leong WF, Yen GG (2008) Pso-based multiobjective optimization with dynamic population size and adaptive local archives. IEEE Transaction on Systems Man and Cybernetics: Part B 38: 1270-1293.
